# Clinical and cost-effectiveness of non-medical prescribing: A systematic review of randomised controlled trials

**DOI:** 10.1371/journal.pone.0193286

**Published:** 2018-03-06

**Authors:** Timothy Noblet, John Marriott, Emma Graham-Clarke, Debra Shirley, Alison Rushton

**Affiliations:** 1 Centre of Precision Rehabilitation for Spinal Pain, School of Sport, Exercise and Rehabilitation Sciences, University of Birmingham, Edgbaston, Birmingham, United Kingdom; 2 Institute of Clinical Sciences, College of Medical and Dental Sciences, University of Birmingham, Edgbaston, Birmingham, United Kingdom; 3 Faculty of Health Sciences, University of Sydney, Sydney, NSW, Australia; Robert Gordon University, UNITED KINGDOM

## Abstract

**Objective:**

To evaluate the clinical and cost-effectiveness of non-medical prescribing (NMP).

**Design:**

Systematic review. Two reviewers independently completed searches, eligibility assessment and assessment of risk of bias.

**Data sources:**

Pre-defined search terms/combinations were utilised to search electronic databases. In addition, hand searches of reference lists, key journals and grey literature were employed alongside consultation with authors/experts.

**Eligibility criteria for included studies:**

Randomised controlled trials (RCTs) evaluating clinical or cost-effectiveness of NMP. Measurements reported on one or more outcome(s) of: pain, function, disability, health, social impact, patient-safety, costs-analysis, quality adjusted life years (QALYs), patient satisfaction, clinician perception of clinical and functional outcomes.

**Results:**

Three RCTs from two countries were included (n = 932 participants) across primary and tertiary care settings. One RCT was assessed as low risk of bias, one as high risk of bias and one as unclear risk of bias. All RCTs evaluated clinical effectiveness with one also evaluating cost-effectiveness. Clinical effectiveness was evaluated using a range of safety and patient-reported outcome measures. Participants demonstrated significant improvement in outcomes when receiving NMP compared to treatment as usual (TAU) in all RCTs. An associated cost analysis showed NMP to be more expensive than TAU (regression coefficient p = 0.0000), however experimental groups generated increased QALYs compared to TAU.

**Conclusion:**

Limited evidence with overall unclear risk of bias exists evaluating clinical and cost-effectiveness of NMP across all professions and clinical settings. GRADE assessment revealed moderate quality evidence. Evidence suggests that NMP is safe and can provide beneficial clinical outcomes. Benefits to the health economy remain unclear, with the cost-effectiveness of NMP assessed by a single pilot RCT of low risk of bias. Adequately powered low risk of bias RCTs evaluating clinical and cost effectiveness are required to evaluate NMP across clinical specialities, professions and settings.

**Registration:**

PROSPERO (CRD42015017212).

## Introduction: Rationale

Non-medical prescribing (NMP) contributes to the effective management of both acute and chronic conditions which require prescription of appropriate medication in a timely manner, without the service users’ needs being affected by health services’ staffing deficiencies, financial concerns or geographical location [1]. It is utilised by a range of professions, with limited consistency regarding definition and terminology internationally [2]. In recent years, the UK government has expanded the scope of NMP that now includes nursing, pharmacy, podiatry, radiography, optometry, physiotherapy and dietetic professions, with the potential to expand further to include paramedicine [3].

With the ever-increasing financial challenges faced by health services, in part due to ageing populations and rising levels of chronic disease, the potential financial efficiencies gained through the use of NMP are of paramount importance [3, 4]. A range of robust studies utilising survey designs have concluded that NMP practice is both safe and appropriate, exhibiting good patient satisfaction [5–9]. Despite this, the implementation of NMP in the UK remains at a relatively slow pace [3]. Although the reasons for this are unclear, it is argued that this is caused by a lack of persuasive high quality evidence demonstrating the clinical and economic benefits of NMP in comparison to current models of healthcare [3]. As demand for healthcare increases, it is likely that policy makers and healthcare departments will become increasingly interested in optimising the skills of all health professionals to streamline patient care [3]. Employing non-medical prescribers within healthcare services has the potential to make savings across a range of health specialties, providing more holistic patient care within an individual profession’s scope of practice [3, 4, 10].

For NMP to become more widely accepted, healthcare managers, clinical care quality and safety agencies, as well as the general public require evidence of the overall value of NMP; through the implementation of services that are patient-centred, improving the quality and safety of patient care, while simultaneously reducing costs and improving efficiency of treatment and patient-outcomes [3, 11]. A robust evaluation of NMP is imperative to ensure quality, and appropriate and efficient use of medicines [12]. The advantages, although anecdotal, are evident in results from case studies and clinical audits which demonstrate that NMP has a good safety record and benefits both patients and clinical services [3, 5]. A recent Cochrane review compared resource utilisation and assessed for non-inferiority in clinical outcome measures and patient reported outcomes of NMP to medical prescribing, concluding that non-medical prescribers provide comparable care across a range of clinical specialties [13]. This systematic review included high risk of bias evidence from controlled trials (Randomised controlled trials (RCTs), cluster-RCTs, controlled before-and-after (CBA) studies and interrupted time series analysis). The future development of NMP across professions internationally is dependent on low risk of bias evidence regarding clinical and cost-effectiveness; without which, it is difficult to demonstrate that NMP offers quality care and patient safety [3]. To date, no systematic review has synthesised this existing evidence.

### Objective

To evaluate the clinical and cost effectiveness of NMP.

## Methods

A systematic review was conducted according to a pre-defined protocol informed by the Cochrane handbook [14–17], and is reported in accordance with the PRISMA statement [17, 18]. The systematic review protocol was registered with PROSPERO (CRD42015017212) to ensure transparency [15, 19]. This article reports objective 1 of the published protocol.

### Eligibility criteria

### Inclusion criteria

#### Studies

Randomised controlled trials (RCTs) or pilot RCTs that evaluated the clinical or cost effectiveness of NMP.

#### Participants

Health service users receiving treatment from non-medical prescribers from any professional group with appropriate authority to prescribe medicines via supplementary or independent prescribing mechanisms [20].

#### Intervention

Non-medical prescribing provided by a professional group with appropriate authority to prescribe medicines via supplementary or independent prescribing mechanisms [20]

#### Comparators

Inter- or intra-profession comparisons of clinical and cost effectiveness, pre and post intervention comparisons of clinical outcomes [14, 21].

#### Outcome Measures

Measurements reported on one or more outcome of: pain, functional impairment, disability, health, social impact, patient safety, associated costs analysis, quality adjusted life years (QALYs), patient satisfaction, clinician perception of clinical and functional outcomes [14].

#### Exclusion criteria

studies not written in English [18].

### Information sources

**Fig 1:**
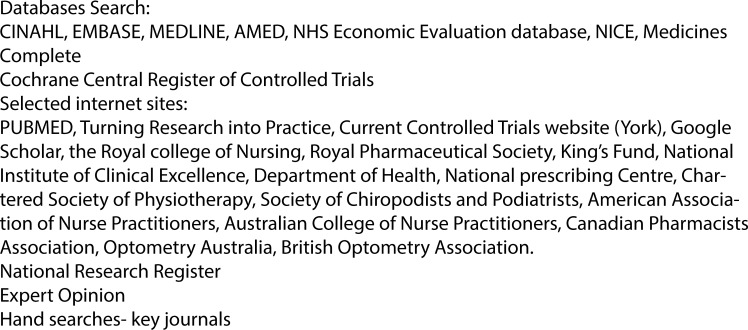
Information sources utilised.

The literature search employed sensitive topic-based strategies designed for each of the sources identified in Fig 1.

### Search

**Fig 2:**
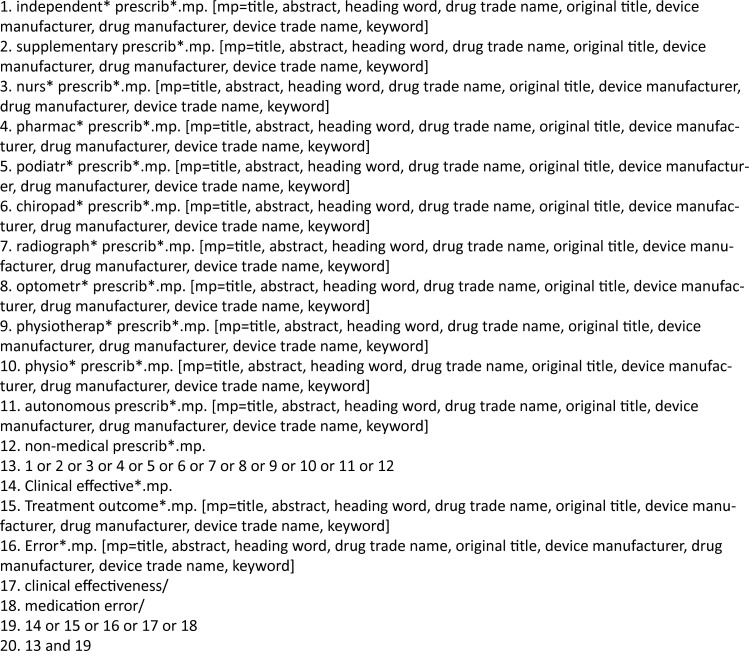
Full electronic search strategy for Medline OvidSP (clinical effectiveness). Originally undertaken: 25^th^ May 2015. Most recently undertaken: 1^st^ November 2016.

Pre-defined search terms and combinations, with database specific standardised vocabulary were employed to ensure all relevant studies were retrieved [14, 21–23]. Fig 2 illustrates an example full electronic search strategy for studies investigating clinical effectiveness in Medline OvidSP. Where a pilot study was identified, the definitive study was sought, or the authors contacted to determine whether further published or unpublished research had been undertaken. The reference lists of the identified literature were searched to ensure no studies were missed [21, 23]. In addition, experts in the area were consulted to detect any further studies [14, 21–23].

### Study selection

Two investigators searched information sources (TN/EGC) and independently assessed studies for inclusion by grading each eligibility criterion. In the event of a selection disagreement a third reviewer (AR, methodological expert) was available to mediate any conflict [19, 22]. Both reviewers independently evaluated studies by title and abstract for potential eligibility. Following discussion between reviewers, if a study could not explicitly be excluded on the basis of its title and abstract, its full text was reviewed [15, 17]. All potentially relevant studies proceeded forward to the review of full text. The two independent reviewers made independent judgements as to whether or not an individual study was included in the review based on the study’s full text fulfilling the eligibility criteria. The numbers of studies included and excluded at the different stages were recorded [14, 19, 21].

### Data collection process

Data extraction was performed by the primary reviewer (TN) and checked and agreed by the secondary reviewer (EGC). Data extraction utilised pre-determined data extraction sheets specific to the review objective which had been piloted, refined and agreed by the researchers prior to use, ensuring that all relevant data were extracted [19, 21]. Any differences were resolved at a consensus meeting of all authors [22], and the third reviewer (AR) checked for consistency and clarity.

### Data items

Study design, profession of prescribers, type of non-medical prescribing, participants (patient groups) and indications, interventions, study settings, timing of assessments, and outcome measures were extracted [14], to allow for assessment of homogeneity [14, 21].

### Risk of bias

Each reviewer independently assessed the internal validity of each included trial using the Cochrane Risk of Bias Tool [14, 24]. This tool was selected as it was developed to specifically assess bias within RCTs [14, 24]. The tool has been evaluated and has been shown to exhibit good inter-rater reliability [25]. Results were tabulated to demonstrate of the risk of bias across included trials [24].

### Summary measures and synthesis of results

An explanation of each included trial’s characteristics and outcome data were tabulated. Within and between studies analyses was undertaken in the context of risk of bias [15, 18].

## Results

### Study selection

**Fig 3:**
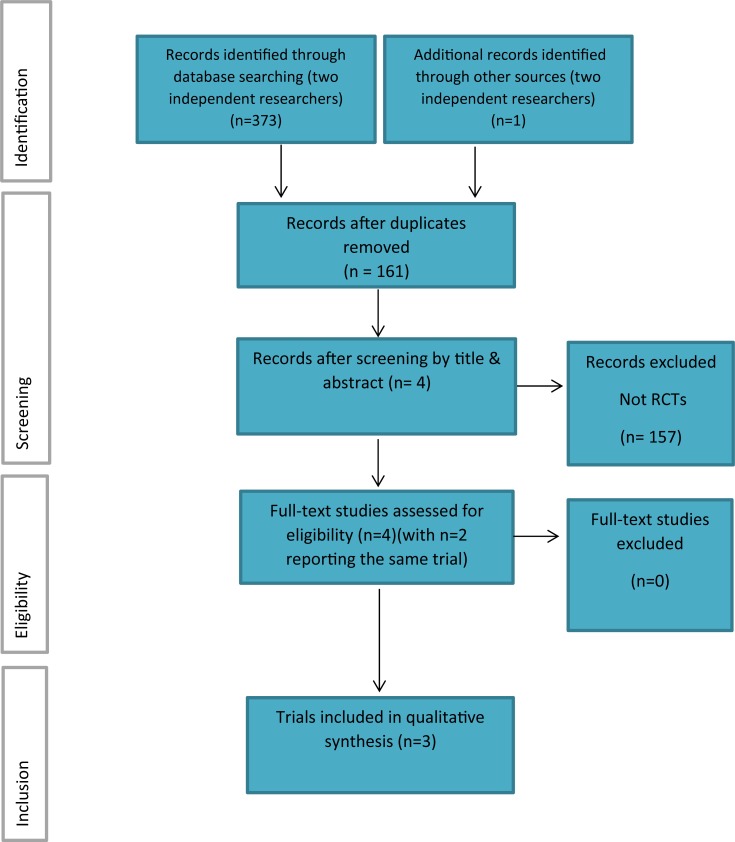
Study selection flow diagram (adapted from Moher et al, 2009)[18].

The search strategy identified 373 potentially relevant studies. Following screening for duplicates, 61 citations remained. No relevant unpublished studies were found and no further studies were identified from the Internet searches, reviews of the national research register or via experts in the field. Reviewing by title and abstract excluded 158 studies that were not RCTs. The full texts of the remaining 3 trials [26–28] were examined in detail and evaluated as meeting the inclusion criteria. A further article [29] retrieved when examining the reference lists of retrieved studies was included as it presented additional data to an included RCT. The data from the two articles were considered as one pilot trial (The PIPPC pilot trial) [26, 29]. Therefore, 3 trials (2 definitive trials and 1 pilot trial) were included (Fig 3). All included trials investigated clinical effectiveness (n = 3) [27–29]; 1 trial investigated cost effectiveness [26]. Fig 3 presents the number of studies at each stage of the selection process. 100% inter-reviewer agreement was achieved following open discussion at each stage. Third reviewer mediation was not required.

### Study characteristics

Study characteristics and descriptive data for the 3 included trials are summarised in Table 1. All 3 trials involved pharmacy as the NMP profession evaluated in the experimental arms of trials. The setting for one trial was the UK [26, 29], and for two was Australia [27, 28]. All included trials compared pharmacist prescribing within a service or specific patient population to usual care.

**Table 1:** Study characteristics of included trials.

Trial	Design	Participants & Indication	Intervention & Setting	Outcome Measures	Between Group Results	Additional Information
The PIPPC Trial (Neilson et al, 2015, Bruhn, 2013)[26, 29]	Pilot RCT: Three Groups: A. Pharmacist medication review plus face-to-face prescribing B. Pharmacist medication review with GP prescribing C. Treatment as usual Recruitment March-June 2010	• Patients >18 years, living independently, receiving regular prescribed medication for pain. • Patients must have received ≥2 acute prescriptions within the preceding 120 days for an analgesic and/or NSAID. • GPs excluded patients with severe mental illness, recent bereavement, alcohol/drug addiction and cancer pain Baseline: A. n = 68 B. mean (SD) age 66.1 (12.1), C. 54.4% female n = 62, age 65.7 (14.2), 46% female n = 63, age 64.9 (11.6), 37% female	A: Face-to-face pharmacist prescribing, with pre-consultation paper-based medication review; patients completed a pain diary. All non-controlled drugs issued via IP. Controlled drugs issued by SP (regulations at the time). B: Pharmacists undertook paper-based medication reviews focussed on pain related prescription medications, implementation by GPs. C: Treatment as usual GP care X6 pharmacist prescribers utilised *Setting*: • GP practices, primary care pharmacies. • UK (Scotland & England)	*Primary Clinical outcome*: SF12v2 Health Utilities Index (data not analysed due to licencing laws) *Secondary Clinical*: CPG HADS *Primary Economic*: Costs associated with: • Intervention (source- PSSRU 2009/2010) • pain related hospitalisation (source- IDS Scotland March 2010) • primary care visits for chronic pain (source- PSSRU 2009/2010) • primary care telephone contacts for chronic pain (source- PSSRU 2009/2010) • OTC pain related medication: Source- BNF 61, March 2011 *Secondary- Effect of pharmacist-led intervention*: QALYs- based on SF-6D	*Clinical outcomes*: SF12v2: no statistical significant difference between groups. CPG: Statistically significant improvement for group A compared to groups B&C for intensity (p = 0.02) but not disability (p = 0.55). HADS: Statistically significant improvement in HADS scores for group A compared to group B&C (A: p = 0.022; D: p = 0.045) *Cost effectiveness outcomes*: *Resource use and costs*: Positive incremental mean cost differences reported for groups A&B compared to C, indicating group A&B interventions are more expensive than group C. *QALYs*: After adjusting for baseline SF-6D scores, baseline costs/controlling for baseline patient characteristics, QALYs for groups A&B were largely unchanged relative to group C.	NMP Qualification: Independent Pharmacist Prescribing Course accredited by the General Pharmaceutical Council, UK. NMP- Pharmacists Independent Prescribing, supplementary prescribing. Exploratory trial to estimate the sample size for full trial- no formal power calculation. Optimal trial size estimated at 780 per group for full study.
Hale et al, 2013[27]	RCT: Two Groups: A. Pharmacist generated medication chart/plan for peri-operative medication/ prescribed VTE prophylaxis. B. TAU. Post consent, patients randomised using computer-generated randomisation in blocks of 10. Independently prepared sealed envelopes containing 1 or 0 then determine allocation. Conducted between June-Sept 2009.	All patients > 18 years, who attended the PAC. Patients were excluded if unable to communicate due to language barrier or undergoing day surgery. Baseline: A. n = 190, mean (mean range) age 57.6 (18–89), 58% male B. n = 194, mean (mean range) age 55.8 (18–86), 59% male	*Intervention*: Group A: Patients seen by a nurse, prescribing pharmacist, RMO and anaesthetist. (Pharmacist prior to RMO). Pharmacist undertook duties as per usual care, plus prescribing. The scope of prescribing: continuing/ withholding regular medications & prescribing VTE prophylaxis according to local & national guidelines. Group B: all 4 professionals consulted in no particular order. Prescribing was the responsibility of the RMO. X1 Pharmacy Prescriber utilised. *Setting*: • X1 Tertiary Hospital • Elective Surgery Preadmissions clinic (PAC) at Princess Alexandria Hospital, Brisbane, Australia.	*Primary clinical outcome*: Frequency of omission & prescribing errors when compared against patient’s medical history. The clinical significance was also analysed. *Secondary clinical outcome*: Appropriateness of VTE prophylaxis prescribing.	*Clinical outcomes*: Significantly less unintended omissions of medications by group A compared to group B. Significantly less prescribing errors involving selection of drug, dose or frequency by group A compared to group B. VTE prophylaxis on admission to the ward approx. 93% group A & 90% group B, revealing no significant difference. No difference in appropriateness of VTE prophylaxis on admission between the two groups.	NMP Qualification: Independent Pharmacist Prescribing Course accredited by the General Pharmaceutical Council, UK. NMP- Pharmacists Independent Prescribing. An amendment was facilitated to the Queensland Health (Drugs and Poisons) Regulation 1996 to enable the qualified pharmacists to prescribe in Queensland, Australia. Power calculations based on pilot data used to calculate sample size.
Marotti et al, 2011[28]	RTC: Three Groups: A. Pharmacist medication history plus supplementary prescribing. B. Pharmacist medication history taking, prescribing through usual process. C. TAU. Blinded computer-generated randomisation. Conducted between Nov 2008- March 2009	All adults (no definition) elective surgery patients excluding orthopaedics. Patients excluded if: no regular medications, unable to provide consent, medications charted at a pre-op clinic appointment, day case. Baseline: A. n = 118, median (IQR) age 64 (47–75), 51% male B. n = 119, median (IQR) age 62 (52–71), 55% male C. n = 118, median (IQR) age 65 (54–75), 49% male	*Intervention*: Groups A&B- pharmacists interviewed patients at the time of admission on day of surgery & documented regular medication list. Group A- the pharmacist prescribed the regular medications on the medication chart via supplementary prescribing. Group C- patients had no interaction with the pharmacist prior to surgery. Medications were charted immediately post-surgery by the medical officer in the normal time frame. *Setting*: • X1 Tertiary Hospital. All surgical units, John Hunter Hospital, Newcastle, NSW, Australia.	*Primary clinical outcome*: The number of medication doses missed inappropriately during the inpatient stay. *Secondary clinical outcome*: Number of medications charted at incorrect dose or frequency. Number of missed medication doses post operatively of significant medications e.g. beta blockers, 3-hydroxy-3-methyl-glutaryl-CoA reductase inhibitors, antiplatelets, anticoagulants.	*Clinical Outcomes*: Significantly reduce number of missed doses per patient during hospital stay for group A (p = 0.02) but not group B compared to group C. Significantly less medications charted at an incorrect dose for Groups A (p<0.001) &B (p = 0.004) compared to group C, with group A having less errors that group B. Significantly less numbers of medications charted at an incorrect frequency by groups A&B compared to group C (p<0.001).	Non-medical prescribing qualification/ credential/ accreditation not disclosed. NMP- Pharmacists supplementary prescribing. No power calculations used to calculate sample size.

IP- Independent Prescribing, SP- Supplementary Prescribing, CPG- Chronic pain grade (CPG), HADS- Hospital Anxiety & Depression Score, PSSRU- Personal Social Services Research Unit, QALYs- Quality-adjusted life years, TAU- Treatment as usual, Venous thromboembolism- VTE, PAC- Pre-admission clinic, ROM- Resident Medical Officer

A total of 932 participants with an age range of 18–89 years, were randomised across the 3 trials. Details regarding the participants’ specific diagnoses were not disclosed. Participants were either: admitted to a tertiary hospital for surgery, involving an overnight stay [27, 28], or received regular prescriptions for medication for chronic pain within a primary care setting [26, 29].

Two trials were completed at single site surgical departments of tertiary hospitals in Australia (Brisbane, Queensland and Newcastle, New South Wales)[27, 28], with a third trial undertaken in primary care across six general practices in the UK (England and Scotland). The type and scope of non-medical prescribing utilised by the pharmacists varied. One trial guided by protocols, used supplementary prescribing to prescribe the patients’ regular medication [28]. One trial used independent prescribing only, where the scope of prescribing was to either continue or withhold regular medications and to prescribe VTE prophylaxis in accordance with local and Australian guidelines [27], and a single trial, owing to regulations in place at the time of study, utilised supplementary prescribing to prescribe controlled drugs and independent prescribing for all other required medications [26, 29].

The prescribing pharmacists in two trials were registered independent pharmacist prescribers having completed an Independent Pharmacist Prescribing Course accredited by the General Pharmaceutical Council, UK [26, 27, 29]. An amendment to the Queensland Health (Drugs and Poisons) Regulation 1996 enabled the qualified pharmacists to prescribe in Queensland, Australia [27]. There was no disclosure of the mechanisms (qualification/credentialing/accreditation) that were required for the pharmacists to undertake legal supplementary prescribing in the trial completed in New South Wales, Australia [28].

### Outcomes: Clinical effectiveness

Primary outcome measures assessing clinical effectiveness varied. Bruhn et al (2013) used the SF12v2 and the Health Utilities Index (HUI). However, because licencing costs were required to score the data, the HUI was not subsequently analysed. Hale et al (2013) and Marotti et al (2011) did not specify a validated patient reported outcome measure, however they analysed the safety of NMP practice, assessing the frequency of omission and prescribing errors when compared against a patient’s medical history, and the number of medication doses inappropriately missed during an inpatient stay respectively.

No comparable secondary outcome measures were used across the three trials. Bruhn et al (2013) assessed pain using the ‘Chronic Pain Grade’ measure and anxiety and depression with the ‘HADS’ (Hospital Anxiety and Depression Scale). The other trials focused on the uses of the medicines prescribed, with one trial examining the appropriateness of VTE prophylaxis prescribing [27] and the other examining the number of medications chartered at an incorrect dose or frequency, and the number of missed doses of specific medications post operatively [28].

### Outcomes: Cost effectiveness

The PIPPC trial evaluated the costs associated with: intervention, pain related hospitalisation, primary care visits for chronic pain, primary care chronic pain related telephone contacts, and prescribed and non-prescribed OTC pain related medicines [26]. Quality-adjusted life years (QALYs) were calculated [26]. The QALYs in the PIPPC trial were generated from the associated costs and analysis of clinical outcomes from the SF-6D (patient reported outcome measure). As this trial was a pilot, the expected value of sample information was calculated to assess whether a definitive trial would be worthwhile.

### Risk of bias

100% inter-reviewer agreement was achieved regarding risk of bias assessment, with no mediation required from the third reviewer. Table 2 provides a summary of the overall risk of bias assessed using the Cochrane Risk of Bias Tool for each included trial. Of the three included trials, one was high risk of bias [27], one unclear [28], and one low risk of bias [26, 29]. Marotti et al (2011) was assessed as unclear risk of bias, as the reviewers were unable to view the registered trial protocol, therefore bias owing to selective outcome reporting remained unclear. Hale et al (2013) was assessed as high risk of bias with the domain ‘blinding of participants’ rated at high risk, whilst all other domains were rated low risk. It was agreed that the weight of this domain to overall risk of bias within the RCT was substantial, as the resident medical officers involved in the trials were aware of the pharmacist prescribing as part of a formal study. Losses to follow-up were reported in all included trials [27, 28]. Across all trials, losses were less than 20% and therefore considered acceptable [30]. The overall risk of bias across trials was evaluated as unclear as 75% of the included studies were rated as low or unclear risk of bias [24].

**Table 2:** Summary assessment of the overall risk of bias for each study.

Study	Domain of risk of bias							Summary within study	Comments on high-risk components
	1	2	3	4	5a	5b	6		
PIPPC Trial [26, 29]	L	L	L	L	L	L	L	**Low** (7)	
Hale et al, 2013[27]	L	L	H	L	L	L	L	Low (6) **High** (1)	One high risk domain: 3 “RMO’s in clinic during the study were aware of the intervention pharmacist’s role, which may have led to an increased number and quality of medication charts prescribed in the control arm.”
Marotti et al, 2011[28]	L	L	L	L	U	U	L	Low (5) **Unclear** (2)	
Overall risk of bias across studies								**Unclear risk of bias**	

**Domain of risk of bias:** 1, sequence generation; 2, allocation concealment; 3, blinding of participants; 4, incomplete outcome data; 5a, short-term selective outcome reporting; 5b, long-term selective outcome reporting 6, other sources of bias.

**Levels of risk of bias:** L, low risk of bias; U, unclear risk of bias; H, high risk of bias

**Summary within study:** Low, low risk of bias for all key risk criteria; Unclear, unclear risk of bias for all key risk criteria; High, high risk of bias for all key risk criteria.

RMO- Resident Medical Officer

### Summary measures and synthesis of results

#### Clinical Effectiveness Outcomes

**SF-12v2:** for functional health and wellbeing from the patient’s perspective, the PIPPC trial[26, 29] at low risk of bias found no significant difference (p = 0.75) between groups.

**Chronic Pain Grade (CPG):** for overall chronic pain severity (pain intensity and pain-related disability), the trial by Bruhn et al (2013) at low risk of bias found significant improvement on the pain intensity subscale (p = 0.02) for the pharmacist experimental prescribing groups when compared to treatment as usual. This improvement was not found for the disability subscale (p = 0.55).

**Hospital Anxiety and Depression Scale (HADS):** for depression, anxiety and emotional distress, the trial by Bruhn et al (2013) at low risk of bias found that both the experimental groups involving prescribing pharmacists were seen to improve significantly more compared to the treatment as usual group (Group A p = 0.022, Group B p = 0.045).

The frequency of omission and prescribing errors: when compared against a patient’s medical history, the trial by Hale et al (2013) which was at high risk of bias found significantly less unintended omissions of medications when prescribed by the pharmacist (p<0.001). There were significantly fewer prescribing errors concerning selection of drug, dose or frequency in the non-medical prescribing group (p<0.001), and significantly less medication orders from the NMP group with at least one constituent of the prescription missing, incorrect or imprecise compared to that of the control group (p<0.001).

Prescription of VTE prophylaxis: the trial by Hale et al (2013) at high risk of bias found no significant difference between the NMP group and the control group (p = 0.29) for the appropriateness of prescription of VTE prophylaxis.

The number of medication doses missed inappropriately during an inpatient stay: the trial by Marotti et al (2011) that had an unclear risk of bias, found a significant difference (p = 0.002) between the pharmacist supplementary prescribing group compared to the pharmacist drug history taking group and the control group for the number of medication doses inappropriately missed during an inpatient stay. The number of drugs charted at the wrong dose and/ or frequency was significantly reduced in the pharmacy history taking group and the pharmacist-prescribing group (p<0.001), compared to that of the control group. The pharmacist-prescribing group were also seen to have fewer dose errors compared to the pharmacy drug history taking group (p = 0.004).

#### Cost Effectiveness Outcomes

**Associated Costs:** the PIPPC trial [26] which had a low risk of bias, found that both pharmacist prescriber-led intervention groups were less costly than TAU based on raw unadjusted mean total costs. Adjustment for variances in baseline costs and controlling for baseline participant characteristics resulted in a positive incremental mean cost difference for both the experimental groups compared to the TAU group. Following adjustments, both pharmacist prescribing and review groups were significantly (regression coefficient p = 0.00) more expensive than usual care secondary to baseline costs.

**Quality-Adjusted Life Years (QALYs):** the PIPPC trial [26] at low risk of bias found for unadjusted data, that both experimental groups generated increased QALYs compared to TAU. Following adjustment for baseline costs, pharmacist-led groups were largely unchanged relative to the TAU (‘pharmacist prescribing’ group, 0.0069 QALYs, <-0.0091 to 0.0229>, ‘pharmacist medication review’ group, 0.0097QALYs, <-0.0054 to 0.0248>), although the adjusted difference in cost was reduced in the prescribing group (£21, from -£124 to £167) and increased in the review group (£75, from -£72 to £221) relative to the TAU group.

### Additional analyses

Meta-analysis was not justified owing to insufficient homogeneity of the outcome measures used across the trials. Although the interventions used across the trials were similar, the low number of trials included compounds the heterogeneity of the outcome measures.

## Discussion

### Summary of evidence

Owing to the low number of included trials and overall unclear risk of bias, recommendations about NMP in the context of its potential clinical and cost-effectiveness are limited. Adequate patient safety and clinical outcomes are key elements in clinical effectiveness required for the valid and ethical use of any clinical intervention. Evidence with an overall unclear risk of bias across the trials investigating the safe practice of NMP on tertiary care surgical wards, indicates that NMP may lead to a significant reduction in omissions and prescribing errors, with the medications prescribed by medical and non-medical prescribers being equally appropriate[27, 28]. Further, the PIPPC pilot trial low risk of bias evidence[29], suggests that NMP is practical, acceptable and leads to improvement in pain outcomes in primary care. However, it is unclear from the PIPPC trial data whether the participants’ improved pain outcomes were due to the changes in medication prescribed by the pharmacists and/or participants’ education regarding optimal timing for administration of the medications. The heterogeneity of the included trials did not allow for meta-analysis. This evidence, when combined with the findings from the previous Cochrane review [31], might indicate that non-medical prescribers can independently optimise medication management for chronic pain as effectively as medical prescribers, and therefore have the potential to effectively support over-stretched medical practitioners working in pain management in primary care.

Embedding a new clinical tool or process into practice often requires explicit economic benefit before it is adopted by a health community [3, 11]. For this reason, it was surprising that only one pilot RCT evaluating the cost-effectiveness of NMP exists[26], even though NMP is now widely practised internationally by a range of health professions. It is important that the results of this trial are interpreted in the context of it being a pilot trial, with the aim to estimate optimal sample size for a definitive trial, not to determine effectiveness. The trial’s results [26], evaluated as having low risk of bias, suggest at first glance that pharmacist prescribing may be more costly than traditional treatment once baseline costs are accounted for (e.g. education costs required for pharmacist prescribers to become qualified, endorsed and registered as non-medical prescribers). However, these baseline costs relate directly to the development of new services that use NMP, where non-medical prescribers do not currently exist and full support for new non-medical prescribers is required. This may be short sighted, reflecting only initial set-up costs, rather than future long-term patient care. As the development, implementation and utilisation of NMP varies across professions internationally, future economic assessment should ensure that both initial and ongoing costs are analysed, establishing economic benchmarks for future comparisons. The SF-6D outcome measure was used to calculate a QALY effect[26], with results indicating that the use of non-medical prescribers generated increased QALYs. However, incomplete data (one third of questionnaires incomplete), possibly owing to participant understanding and the complexity of the measure [26] may have had significant influence on the outcomes and should be considered further prior to the design of an adequately powered definitive trial.

Comparison of the results from this review with the wider literature is difficult, as no RCTs in addition to those included in the present review have been undertaken, and there are no previous systematic reviews. The majority of research has concentrated on reporting the experiences of stakeholders and has not used validated outcome measures to investigate cause and effect relationships related to the uses of NMP[26]. The potential benefits of NMP in terms of clinical and cost-effectiveness are illustrated by the included trials, however the deficit of low risk of bias RCTs across professions, specialties and settings, highlights the need for adequately powered low risk of bias RCTs to inform both clinical and cost-effectiveness across important outcome measures. In order to enhance the quality and comparability of future RCTs, the development of a minimum data-set of important outcome measures for the assessment of NMP would be beneficial, providing healthcare managers, clinical care quality and safety agencies as well as the general public with the require evidence needed to evaluate the overall value of NMP.

### Strengths and limitations of the review

This is the first systematic review to synthesise the existing evidence using rigorous methods to provide clarity of the level and quality of existing evidence. Evaluation of the evidence using GRADE (the Grading of Recommendations Assessment, Development and Evaluation system) assessment revealed moderate quality evidence for both the clinical and cost effectiveness of NMP (Table 3)[32]. However, the limited number of trials available for inclusion and overall unclear risk of bias of the included trials limits the external validity of the review. Each trial used different outcome measures limiting scope for meta-analysis, due to limited homogeneity. Only NMP by pharmacists was investigated limiting generalisability across all professions. Limitations in the diversity of the included nations, specialties, methods of NMP (independent versus supplementary) and the nature of the use of NMP were evident between the included studies, resulting in high heterogeneity, limiting their comparability and ability to make generalisations.

**Table 3:** GRADE assessment summary.

**Clinical effectiveness: NMP V TAU**		Sample population size **(n) = 932**	
**Trials Contributing:** Bruhn et al, 2013 [29]; Hale et al, 2013 [27]; Marotti et al, 2011 [28]			
Type of evidence	+4	RCTs	
Quality	–1	Problem with 1 element, blinding not utilised in any trial	
Consistency	0	Most studies show similar results	
Directness	–1	Problem with 1 element, Problem with 1 element, difficulty generalising across all specialties, professions, locations, health-sectors, internationally	
Effect size	+1	<0.5 for all studies	
**Total**	**3**	**Moderate Quality**	
**Cost-effectiveness: NMP V TAU**		Sample population size **(n) = 193**	
**Trials Contributing:** Neilson et al, 2015 [26]			
Type of evidence	+4	RCTs	
Quality	–1	Problem with 1 element, Other methodological concerns: incomplete inclusion and used of SF12 data	
Consistency	0	Only x1 trial included	
Directness	–1	Problem with 1 element, Problem with 1 element, difficulty generalising across all specialties, professions, locations, health-sectors, internationally	
Effect size	+1	<0.5 for all studies	
**Total**	**3**	**Moderate Quality**	

## Conclusions

This systematic review has identified limited evidence with moderate quality and unclear risk of bias evaluating the clinical effectiveness of NMP across all professions and clinical settings. Three trials have shown significant results indicating that NMP is safe and can provide effective clinical outcomes for patients. The benefit to the health economy remains unclear, with the cost-effectiveness of NMP assessed by a single pilot RCT that, although at low risk of bias, by its nature was not powered to evaluate cost-effectiveness. Adequately powered low risk of bias RCTs, evaluating safety, quality, appropriateness of care and economic benefit across a range of clinical professions, specialties and settings is urgently required. Evidence from future RCTs can then be used to inform politicians, policy makers, clinicians and healthcare managers when considering the utilisation of NMP in the planning and provision of future quality and effective healthcare services[3, 4]. The development of a minimum data-set of outcome measures is required to ensure homogeneity/ comparability of data when analysing and assessing non-medical prescribing within and across individual clinical fields, professions, and across international healthcare boundaries.

## Supporting information

S1 Fig PRISMA Checklist. (DOCX)
